# Autosomal Microsatellite Investigation Reveals Multiple Genetic Components of the Highlanders from Thailand

**DOI:** 10.3390/genes12030383

**Published:** 2021-03-08

**Authors:** Aornpriya Mawan, Nonglak Prakhun, Kanha Muisuk, Suparat Srithawong, Metawee Srikummool, Jatupol Kampuansai, Rasmi Shoocongdej, Angkhana Inta, Sukhum Ruangchai, Wibhu Kutanan

**Affiliations:** 1 Department of Biology, Faculty of Science, Khon Kaen University, Khon Kaen 40002, Thailand; a.mawan@kkumail.com (A.M.); nonglak.p@kkumail.com (N.P.); Suparat2117@gmail.com (S.S.); 2 Department of Forensic Medicine, Faculty of Medicine, Khon Kaen University, Khon Kaen 40002, Thailand; mkanha@kku.ac.th; 3 Department of Biochemistry, Faculty of Medical Science, Naresuan University, Phitsanulok 65000, Thailand; metaweesr@nu.ac.th; 4 Department of Biology, Faculty of Science, Chiang Mai University, Chiang Mai 50202, Thailand; Jatupol_K@hotmail.com (J.K.); aungkanainta@hotmail.com (A.I.); 5 Research Center in Bioresources for Agriculture, Industry and Medicine, Chiang Mai University, Chiang Mai 50202, Thailand; 6 Department of Archaeology, Faculty of Archaeology, Silpakorn University, Bangkok 10200, Thailand; rasmi13@hotmail.com; 7 Department of Physics, Faculty of Science, Khon Kaen University, Khon Kaen 40002, Thailand; sukhrua@kku.ac.th

**Keywords:** hill tribes, microsatellites, Hmong-Mien, Thailand, forensic database

## Abstract

The hill tribes of northern Thailand comprise nine officially recognized groups: the Austroasiatic-speaking (AA) Khmu, Htin and Lawa; the Hmong-Mien-speaking (HM) IuMien and Hmong; and the Sino-Tibetan-speaking (ST) Akha, Karen, Lahu and Lisu. Except the Lawa, the rest of the hill tribes migrated into their present habitats only very recently. The Thai hill tribes were of much interest to research groups focusing on study of cultural and genetic variation because of their unique languages and cultures. So far, there have been several genetic studies of the Thai hill tribes. However, complete forensic microsatellite database of the Thai hill tribes is still lacking. To construct such database, we newly generated 654 genotypes of 15 microsatellites commonly used in forensic investigation that belong to all the nine hill tribes and also non-hill tribe highlanders from northern Thailand. We also combined 329 genotypes from previous studies of northern Thai populations bringing to a total of 983 genotypes, which were then subjected to genetic structure and population relationships analyses. Our overall results indicated homogenous genetic structure within the HM- and Tai-Kadai (TK)-speaking groups, large genetic divergence of the HM-speaking Hmong but not IuMien from the other Thai groups, and genetic heterogeneity within the ST- and AA-speaking groups, reflecting different population interactions and admixtures. In addition to establishing genetic relationships within and among these populations, our finding, which provides a more complete picture of the forensic microsatellite database of the multiple Thai highland dwellers, would not only serve to expand and strengthen forensic investigation in Thailand, but would also benefit its neighboring countries of Laos and Myanmar, from which many of the Thai hill tribes originated and where large populations of these ethnic groups still reside.

## 1. Introduction

**Figure 1:**
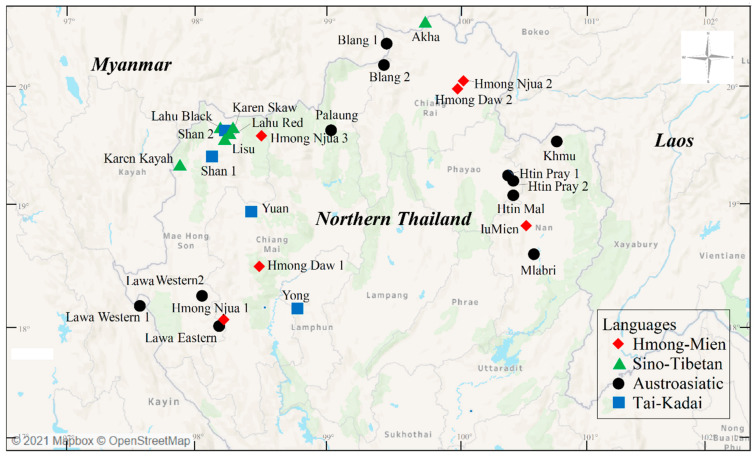
Map of sampling locations. There are 21 populations sampled in present study, together with 6 populations in previous studies. Red diamonds, green triangles, black circles, and blue squares represent Hmong-Mien (HM)-, Sino-Tibetan (ST)-, Austroasiatic (AA)- and Tai-Kadai (TK)-speaking populations, respectively.

Northern and western Thailand are geographically characterized by several small river plains separated by steep mountainous ranges. These areas share borders with Myanmar to the west and with Laos to the east (Figure 1). The many ethnicities inhabiting these regions can be categorized into two groups: the lowlanders and highlanders (consisting of hill tribes and non-hill tribes). The Tai-Kadai (TK)-speaking populations, e.g., Yuan, constitute the major groups who live in lowland areas, while most of the highlanders speak Austroasiatic (AA), Sino-Tibetan (ST) and Hmong-Mien (HM) languages. With the census size of ~700,000 people, there are nine officially recognized hill tribes: the AA-speaking Khmu, Htin and Lawa; the HM-speaking IuMien and Hmong; and the ST-speaking Akha, Karen, Lahu and Lisu [1,2]. The Karen (with several subgroups, e.g., Pwo, Skaw and Kayah) and Hmong are the major hill tribes with a respective population of ~444,100 and ~92,000 people, while the Lawa (~8000 for Lawa Eastern and ~8500 for Lawa Western) and the Htin subgroup Mal (~3500) make up a small proportion of the hill tribes [3]. Except the Lawa, most of the hill tribes migrated from neighboring countries of Myanmar, Laos and southern China to present-day Thailand ~200 years ago (ya) via different routes and due to different reasons [1,2]. In addition to the nine officially recognized hill tribes, the AA-speaking Mlabri, Palaung and Blang, and the TK-speaking Shan are highland dwellers with no official recognition as hill tribes. The numbers of Mlabri, Palaung, Blang and Shan speakers in Thailand are ~400, ~5000, ~1200 and ~95,000, respectively [3].

In socio-economic perspective, after settlements in Thailand, the hill tribes have continued to be a disadvantaged and vulnerable group in Thai society, being largely dependent on agriculture for income and employment. In the past, there were reports of high level of opium addiction [4] and opium cultivation in some hill tribes [5,6], although nowadays the opium problem has largely been resolved. Some of the hill tribes still live in small villages located at uneasily accessible, high-altitude, dense forests along the Thailand-Myanmar and Thailand-Laos borders, but their communities that span transnational borders have still contacted for socio-economic activities. Therefore, migration, illegal trading as well as deforestation are cross-border issues affecting all three countries [7].

In anthropological perspective, as a result of their living in a small, remote and isolated area, linguistic and cultural variations of the Thai hill tribes are of interest to several scholars conducting anthropological and ecological studies [8,9]. Different post marital residence patterns are found in the Thai hill tribes, making it a unique case for studying the effects of cultural practices on the genetic diversity of the populations. These residence patterns vary among the hill tribes with the Hmong, IuMien, Lisu, Lawa, Khmu and Akha practicing patrilocality (i.e., following marriage, the woman moves to the residence of the man), while the Karen, Htin and Lahu are matrilocal (i.e., the man moves to the residence of the woman). To study an effect of patrilocality vs. matrilocality on genetic variation patterns, mitochondrial (mt) DNA vs. Y chromosome variation is commonly utilized and the first such study was carried out on the Thai hill tribes [8], with the variation further investigated in subsequent studies [10,11,12]. Apart from investigation of their genetic variations, previous genetic studies also paid attention to measuring sex-specific differences in migration rates [10], genetic admixture among the AA-speaking Lawa and northern Thai TK groups [13] and predicting ancestral cultural practices [12]. In addition, genetic relationships within and among the different hill tribes have been established. Previous autosomal short tandem repeats (STRs) indicated genetic distinction of the Khmu, Htin and Karen (Skaw, Pwo and Paduang) [14] and admixed structure of the Kayah [15] while autosomal SNPs studies indicate shared genetic ancestry among the hill tribes corresponding with their linguistic affiliations [16], except the Karen who showed affinity with the AA-speaking groups [17,18]. 

Despite a number of intensive studies on the Thai hill tribes, none had reported their complete genetic data. To build a genetic database for these populations, i.e., Hmong (Hmong Daw and Hmong Njua), IuMien, Akha, Lahu (Lahu Black and Lahu Red), Lisu, Karen (Karen Skaw and Karen Kayah), Lawa (Lawa Eastern and Lawa Western), Khmu and Htin (Htin Mal and Htin Pray) (Table 1 and Figure 1), we generated new data of 15 autosomal STRs in the AmpFLSTR Identifiler panel (Applied Biosystems, Foster City, CA, USA). This set of markers shows many advantages in both forensic and population genetics, e.g., its high polymorphisms, mutations in microsatellites accumulate with drift that do not subject to natural selection, and informativeness to differentiate among recently diverged populations [19]. Data on the non-hill tribe highlanders of the TK-speaking Shan, and the AA-speaking Blang, Palaung and Mlabri were also generated (Table 1 and Figure 1). The results of our work would serve as forensic database of the Thai highlanders in Thailand. Home to large populations of these ethnicities, Thailand’s neighbors of Myanmar and Laos would also benefit from our finding, facilitating cross-border cooperation in forensic investigation. We here established not only the complete allelic frequency of forensic STRs of all hill tribe groups, but also explored the genetic diversity, migration and demographic history of the highlanders, including the hill tribes and non-hill tribes of northern Thailand.

## 2. Materials and Methods

### 2.1. Samples, DNA Amplification and STR Typing

Most genomic DNA samples of the Hmong Daw, Hmong Njua, IuMien, Lahu Black, Lahu Red, Lisu, Karen Skaw, Lawa Western 1, Khmu, Htin Mal, Htin Pray 1, Htin Pray 2, Mlabri, Palaung, Blang and Shan 2 were from previous studies [12,14,20], while those of the Akha were newly collected buccal samples obtained with written informed consent and with ethical approval from Khon Kaen University (Protocol Number HE622027). To recruit the samples, all volunteers were interviewed for individual history and screened for unrelated at least two generations and obtained with written and signed informed consent. We extracted DNA using the Gentra Puregene Buccal Cell Kit (Qiagen, Germany) according to the manufacturer’s directions. Fifteen autosomal STR loci, i.e., *D8S1179*, *D21S11*, *D7S820*, *CSF1PO*, *D3S1358*, *TH01*, *D13S317*, *D16S539*, *vWA*, *TPOX*, *D18S51*, *D5S818*, *FGA*, *D19S433* and *D2S1338* of a total of 654 samples were amplified using the commercial AmpFLSTR Identifiler kit, according to the manufacturer’s protocol. The amplicons were genotyped by multi-capillary electrophoresis on an ABI3130 DNA sequencer (Applied Biosystems) and the allele calling was performed by the Gene Mapper software Version 3.2.1 (Applied Biosystems). For genetic comparison analyses, we also retrieved 329 genotypic data of the Karen Kayah, Lawa Western 2, Lawa Eastern, Shan 1, Yuan and Yong from the previous studies [15,21]. General information about the studied populations are shown in Table 1 and Figure 1.

### 2.2. Statistical Analyses

We calculated genetic diversity indices for each locus and population, i.e., the observed heterozygosity (*H_O_*), expected heterozygosity (*H_E_*), average *H_E_*, number of alleles, gene diversity (GD) and standard deviation (SD), allele frequency and Hardy-Weinberg *p*-value (HWE) using Arlequin v.3.5.3.1 [22]. Several forensic parameters, i.e., power of discrimination (PD), matching probability (MP), polymorphic information content (PIC), power of exclusion (PE) and typical paternity index (TPI) as well as the combined PD (CPD), combined MP (CMP) and combined PE (CPE) were calculated by the Excel Power Stats spreadsheet [23].

Arlequin was also used to perform analysis of molecular variance (AMOVA) [24] for the genetic variance at the three hierarchical subdivisions within individuals of a population, among populations within a group, and among groups of populations (according to linguistic classification), and the genetic distance (*F_ST_*) matrix between pairs of populations based on the number of different alleles. The *F_ST_* matrix was plotted in three dimensions by means of multidimensional scaling (MDS) using Statistica v.10 demo (StatSoft, Inc., Tulsa, OK, USA). The R software was used to construct heat plots of the *Φ*_st_ distance matrix and MDS [25].

To identify genetic structure and population clustering, the model-based method as implemented in STRUCTURE version 2.3.4 was used with the following three main parameters: correlated allele frequencies, admixture and assistance of sampling locations (LOCPRIOR model) [26,27,28]. The number of cluster (*K*) was predefined from 2 to 10; ten replications were run for each *K* with burn-in length of 100,000 iterations followed by 200,000 iterations. In order to identify the optimal *K* value in the data, the STRUCTURE outputs were combined to compute a second-order rate of change logarithmic probability between subsequent *K* values (△*K*) [29] by STRUCTURE Harvester [30]. To validate the dynamic procedure identifying the optimal similarity threshold for each value of *K*, CLUMPAK [31] was used to produce a single-set result from 10 replications of STRUCTURE outputs; outputs from CLUMPAK were graphically modified by DISTRUCT [32].

To obtain a broader picture of population relationships within Southeast Asia, we included publicly available STR frequency from relevant populations [15,21,33,34,35,36,37,38,39,40,41,42,43,44,45,46,47,48,49,50,51,52,53,54,55,56,57] and a neighbor-joining tree (NJ) based on *F_st_* computation by allele frequency from 13 STRs of the FBI Laboratory’s Combined DNA Index System (CODIS) was carried out using POPTREE v.2 [58].

## 3. Results

### 3.1. Genetic Diversity and Forensic Parameters

A total of 654 individual raw genotypes are provided in Table S1. The total gene diversity of the combined hill tribes was 0.767 ± 0.385 while that in individual populations of both the hill tribes and non-hill tribes ranged from 0.707 ± 0.363 in the Lahu Red to 0.788 ± 0.400 in the Blang1 (Table 1). Interestingly, the Mlabri show an extremely reduced genetic diversity with gene diversity of 0.547 ± 0.288 (Table 1), no variation at *TPOX* (*H_E_* = 0) and only 51 alleles in total.

For forensic purpose, loci departure from the HWE, average *H_E_*, total alleles, GD and forensic parameters (CMP, CPE and CPD) of the 27 individual populations are shown in Table 1. There are seven loci that depart from the HWE even after applying Bonferoni adjustment (Table 1). To present an allelic frequency table for the 15 STR loci, we combined data from the 19 populations of all nine hill tribes, i.e., Hmong, IuMien, Htin, Khmu, Lawa, Karen, Lahu, Lisu and Akha into one allelic frequency table (Table S2). We also generated an allelic frequency table of all 13 highlanders, in which information on the non-hill tribes of Shan, Mlabri, Palaung and Blang are included (Table S3). In addition, individual allelic frequency tables of each ethnolinguistic group: Akha, Lahu, Karen, Lisu, Hmong, IuMien, Mlabri, Htin, Khmu, Lawa, Palaung, Blang and Shan are presented in Tables S4–S16, respectively. For the allelic frequency table of the combined hill tribe data (Table S2), there are a total of 177 alleles, varying from 7 alleles at *TH01* to 21 alleles at *FGA* (Table S2); their allele frequencies vary from 0.001 to 0.512. The lowest *H_E_* is observed at *TPOX* (0.624), while the highest *H_E_* is the *FGA* (0.884), in agreement with other Thai [49,50,59] and East Asian populations [39,40,55,56]. The PIC and MP range from 0.566 (*TPOX*) to 0.867 (*FGA*) and from 0.027 (*FGA*) to 0.191 (*TPOX*), respectively. The PD ranges from 0.809 (*TPOX*) to 0.973 (*FGA*), with a value of 0.99999999 for the combined PD. The PE ranges from 0.268 (*TPOX*) to 0.633 (*FGA*), with a combined PE value of 0.999967. The allelic frequency table of the combined highlander data shows a total of 191 alleles, varying from 8 alleles at *TH01* and *D13S317* to 21 alleles at *FGA* (Table S3). The lowest *H_E_* is observed at *TPOX* (0.611), while the highest *H_E_* at the *FGA* (0.886). The PIC and MP range from 0.559 (*TPOX*) to 0.871 (*FGA*) and from 0.026 (*FGA*) to 0.197 (*TPOX*), respectively. The PD ranges from 0.803 (*TPOX*) to 0.974 (*FGA*), with a value of 0.99999999 for the combined PD. The PE ranges from 0.248 (*TPOX*) to 0.626 (*FGA*), with a combined PE value of 0.999953.

### 3.2. Genetic Relationship and Genetic Structure

**Figure 2:**
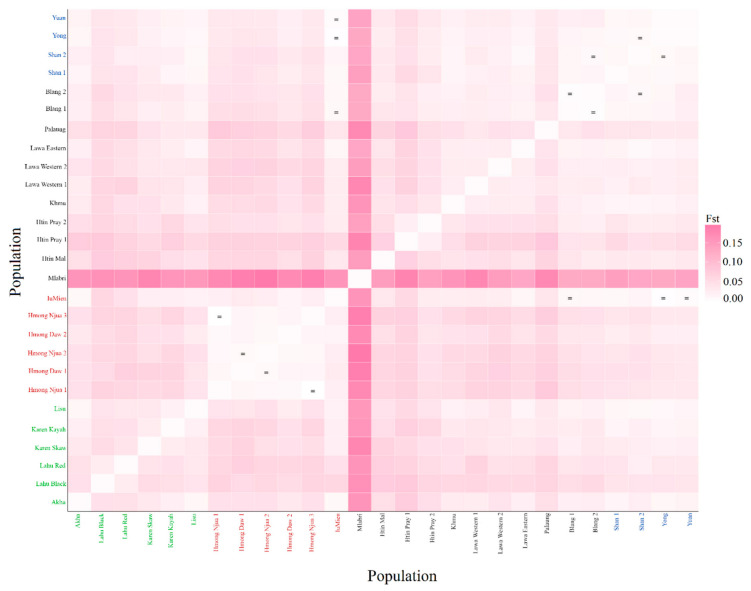
Heat plot of *F_st_* values between total 27 populations. Population names are color-coded according to language family; red, green, black, and blue represent HM-, ST-, AA-, and TK-speaking populations, respectively.

**Figure 3:**
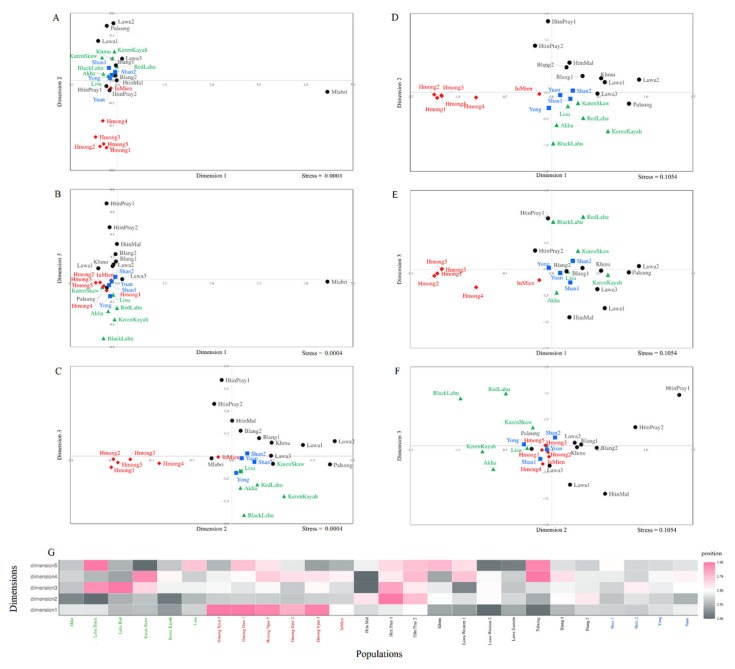
The three-dimensional MDS plot of dimension 1 vs. 2 (**A**), 1 vs. 3 (**B**) and 2 vs. 3 (**C**) of total 27 populations. The three-dimensional MDS plot of dimension 1 vs. 2 (**D**), 1 vs. 3 (**E**) and 2 vs. 3 (**F**) of 26 populations, after excluding Mlabri. The heat plot of standardized values of MDS with five dimensions (**G**). Red diamonds, green triangles, black circles, and blue squares represent HM-, ST-, AA-, and TK-speaking populations, respectively.

Pairwise genetic distances are a measure of genetic relationship among populations. Among 351 pairwise comparisons, there are 343 pairs (97.72%) with statistical differences and eight pairs without significant differences (*p* > 0.05) (Figure 2). The AA-speaking Mlabri shows large differences from the other populations in the heat plots of the *F_st_* values, while the Htin Pray, Htin Mal and Palaung are the next most differentiated AA populations from the other groups. The five homogenous Hmong populations show a genetic difference from the other populations, while the IuMien is genetically more similar to the others than are the Hmong populations. For the ST-speaking populations, both the Lahu Red and Lahu Black are different from the Akha, Lisu and Karen who are rather closely related to the TK or AA groups (Figure 2). We further visualize the population relationships based on the *F*_st_ distance matrix with MDS analysis. The MDS plot for three dimensions indicates genetic distinction of the Mlabri and Hmong populations in dimension 1 and 2 (Figure 3A–C), and after the removal of data on the Mlabri population, a three-dimension MDS for the remaining 26 populations has an acceptable stress value with the MDS showing population clustering according to language family, albeit with some overlapping between them. Located along the edges of the plot, the five Hmong populations (of Hmong Daw and Hmong Njua) are quite distinct from all other groups (Figure 3D–F), whereas the IuMien population is more similar to the other groups than to the Hmong groups; the IuMien overlaps with the TK, most of the AA groups and some ST groups (Akha and Lisu), all clustered in the center of the plot (Figure 3D,E). The AA-speaking Htin Mal and Htin Pray and the ST-speaking Lahu are more spread out, indicating their genetic divergences (Figure 3D–F). Interestingly, the AA-speaking Palaung is closer to the cluster of the ST than the other AA populations (Figure 3D–F). The heat plot of the MDS indicates genetic heterogeneity of the AA- and ST-speaking populations and genetic homogeneity of the HM- and TK-speaking populations. However, within the HM groups, the Hmong and IuMien are genetically different (Figure 3G). In general, the pattern of population clustering is similar to the Y chromosome and mtDNA results from the previous study [12].

**Figure 4:**
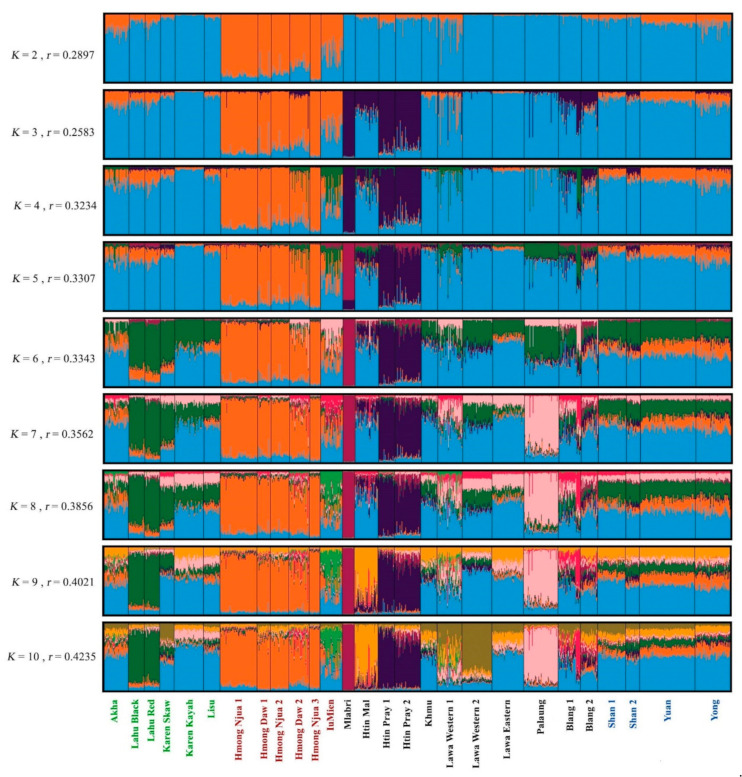
The STRUCTURE result from *K* = 2 to 10. Each individual population is represented by a single column divided into segments whose size and color correspond to the relative proportion of a particular cluster. Populations are separated by black lines. Population names are color-coded according to language family; red, green, black, and blue represent HM-, ST-, AA-, and TK-speaking populations, respectively. *K* and *r* are the number of clusters and parameter which estimates the informativeness of the sampling location data, respectively.

To elucidate a cryptic population structure and relationship, a model-based clustering algorithm, implemented in STRUCTURE with the assistance of a sampling information model, was employed in which different consecutive clusters (*K*) were run from 2 to 10 (Figure 4). The number of *K* at 3 and 6 is the two most suitable △*K* to describe sub-structuring of the studied populations (Figure S1). At *K* = 3, the first cluster detected is in the HM-speaking populations and is represented by the orange color; the second cluster (dark purple) appears in the AA-speaking Mlabri and Htin, while the third cluster (light blue) is predominant in the remaining populations. At *K* = 6, the Mlabri split from the Htin and occupy their own light purple component, while the Htin Mal and Pray share the dark purple component but the former has another major light blue source; the ST-speaking populations are separated into two groups, i.e., a group of Black Lahu, Red Lahu and Karen Skaw who show dark green as their major component and the other group of Akha, Lisu and Karen Kayah who predominantly show light blue, which is similar to the profile of the TK- and AA-speaking Palaungic populations (Lawa, Palaung and Blang); and the IuMien show the minor pink component that also occurs in the AA-speaking Palaungic populations. Notably, although the Khmu, Mlabri and Htin are ethnolinguistically closely related, the Khmu genetic component is light blue which is distinct from their ethnolinguistic relative. Although increasing *K* values are associated with lower delta *K* values, further new components and additional cryptic population structure could emerge. At *K* = 10, the Lahu, Hmong, IuMien, Htin Mal, Htin Pray, Mlabri, Lawa Western and Palaung exhibit their own genetic structures, while the other groups share the common component (Figure 4).

Overall, the HM-speaking Hmong populations show genetic homogeneities within their own groups and have genetic difference from the other hill tribes and comparative northern Thai groups, whereas the HM-speaking IuMien have a broad sharing profile with both the Hmong and other TK, AA and ST populations. The AA-speaking hill tribes exhibit highest within-group genetic heterogeneities with at least three components emerging. Within the ST-speaking populations, both Lahu populations are genetically similar and both diverge from the other groups.

### 3.3. AMOVA Results

The AMOVA results indicate that the variation among populations accounts for 3.91% (*p* < 0.05) (Table 2). The genetic variation among the four language families (HM, ST, AA and TK) is much smaller (1.06%) (*p* < 0.05) than the variation among populations assigned to each group (3.11%) (*p* < 0.05), indicating that language families do not correspond to the genetic structures of these populations. The AA group shows the greatest genetic heterogeneity among populations (4.93%, *p* < 0.05), followed by the ST (3.31%, *p* < 0.05) and HM groups (1.39%, *p* < 0.05), while the TK group shows the lowest among-population variation (0.54%, *p* < 0.05). Although the variation within group among the AA populations is lower when the Mlabri is excluded (3.73%; *p* < 0.05), the AA group remains showing the greatest genetic heterogeneity.

Genetic variation between pairs of the linguistic groups shows significant differences among the groups in almost all comparisons, except in the pairs of AA vs. TK and ST vs. TK, further supporting a close relationship between the TK and other groups. High variations observed between the HM and other groups indicate their genetic distinction from the other linguistic groups. However, variation between the groups is lower than that among the populations within the same groups.

### 3.4. Asian Phylogenetic Tree

**Figure 5:**
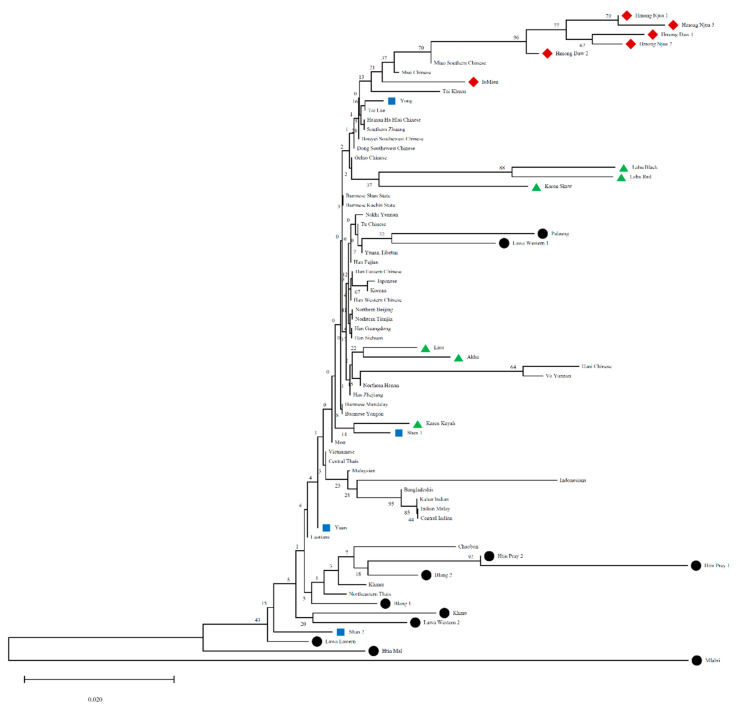
Neighbor-joining tree constructed from *F_ST_* genetic distance based on the allelic frequencies of 13 STR loci among total 69 populations. The different symbols indicate the studied populations from northern Thailand that were used in previous analyses. Red diamonds, green triangles, black circles, and blue squares represent HM-, ST-, AA-, and TK-speaking populations, respectively.

To get a clearer picture on genetic relatedness of the Thai hill tribes with other Asian populations, we constructed a neighbor-joining (NJ) tree based on *F_st_* computation by allele frequency of 13 CODIS STR loci (Figure 5). With greatest divergence of the Mlabri, consistent with other results (Figure 2, Figure 3 and Figure 4), the Mlabri are clustered with the other AA populations from Thailand and this cluster is close to South Asian and other Southeast Asian populations, e.g., Vietnamese, Laotian, Indonesian, except the populations from Myanmar. The populations from Myanmar and East Asia, e.g., China, Japan and Korea are clustered in the same clade that includes the ST-speaking Akha and Lisu and AA-speaking Palaung and Lawa Western 1 from Thailand. All of the Thai Hmong and IuMien populations are clustered with the southern Chinese populations and the TK-speaking populations from Thailand who migrated from southern China. In general, we found that the AA-speaking populations and other populations who were previously reported mixing with the AA groups from Southeast Asia, e.g., central Thai and Indonesian are closer to the South Asian groups, whereas the HM- and ST-speaking populations from Thailand are closer to the Southern Chinese and East Asian.

## 4. Discussion

According to previous maternal and paternal genetic studies of the hill tribes in Thailand, postmarital residence pattern has been shown to influence genetic variation in the Thai hill tribes [8,10,11,12] and previous autosomal SNPs studies indicated shared genetic ancestry among these groups corresponding with their linguistic affiliations [16], with an exception of the Karen who showed affinity with the AA-speaking groups [17,18]. Despite much research on the genetics of the Thai hill tribes, complete forensic database had not been constructed yet. In addition, not all hill tribes were subjected to intensive genetic investigation with the ST-speaking Akha population being less investigated than the other groups. Here, we reported new data on autosomal STRs, that are commonly used for forensic purpose, of all highland dwelling minorities of northern Thailand, hill tribes and non-hill tribes alike. Overall, the results on forensic parameters of all loci indicate that this set of markers is sufficiently informative for personal identification and paternity testing. These 15 loci can distinguish the hill tribe samples from one another with a probability of 99.999999%. Among the nine officially recognized hill tribes, i.e., the AA-speaking Lawa (Western and Eastern), Htin (Mal and Pray) and Khmu; the HM-speaking Hmong (Daw and Njua) and IuMien; and the ST-speaking Karen (Kayah and Skaw), Lahu (Black and Red), Akha, and Lisu, there are four genetically classified groups. All subgroups of the Hmong, Lahu and Htin stand out from the other populations (Figure 2 and Figure 3), while the remaining populations show multiple ancestries suggesting that they might have more interactions with other groups (Figure 4). In addition, although the Palaung is not officially classified as the hill tribe, their differentiation from the other AA groups is observed. Here, we focus to discuss the Hmong, Lahu, Htin and Palaung and also other related groups. 

### 4.1. The Genetic Structure of the Hmong and Their Linguistic Relative, IuMien

There are 35 Hmongic and four Mienic languages within the HM language family distributed across China, northern Vietnam, northern Laos and northern Thailand [60], with the Hmongic and Mienic languages distinct from each other based on a linguistic study [60]. The homelands of the Hmong and IuMien are in southeastern China, from which the Hmong migrated into Thailand through Laos in the second half of the 19th century A.D., while the IuMien started to migrate southwards to Vietnam in the 13th century A.D., entering Thailand about 200 ya [1,10]. The Hmong are the most differentiated group with distinct genetic structure in the STRUCTRE result starting from *K* = 2 (Figure 4) and the MDS result (Figure 3). Striking genetic divergence of the Hmong is also supported by the mtDNA and Y chromosomal results [12]. Specific mtDNA lineages (B5a1c1a* and B5a1c1a1) and prevalent Y chromosomal haplogroups: O2a2a1a2a1a2 (O-N5) and C-F845 to the Thai Hmong also reflect the unique genetic structure in this population [12]. However, the heat plot of pairwise genetic distance (Figure 2) and AMOVA results (Table 2) indicate genetic homogeneity within the Hmong groups. Genetic divergence of the Hmong from other Thai populations as well as their homogenous genetic structure may reflect cultural isolation. Hmong communities have strong connections and they prefer to marry within their group or other Hmong groups and rarely intermarry other hill tribes because intramarriage can extend their clans and can provide greater opportunity for courtship in a village [1,61].

Apart from their genetic distinction from all other Thai groups, the Thai Hmong are genetically distinct from the IuMien who stand out much less in their genetic structure. The location of the IuMien in the center of the MDS plot (Figure 3), non-significant difference in genetic distance values with many populations (Figure 2) and multiples ancestries shown in the STRUCTRE result from *K* = 6 (Figure 4) indicate close genetic relatedness of the IuMien with several populations, reflecting more contact with them. Consistent with the mtDNA and Y chromosomal results [12], the mixed ancestry of the IuMien can be explained by their culture of adoption. Their ethnographic accounts from the 1960s suggest that around 10%–15% of adult Mien have been adopted from other ethnic groups of both highland and lowland in order to increase the size of their household thereby increasing the family’s influence [62]. Some works reported the percentage of adopted individuals to be about 20% [1,10,63]. Another possible reason for driven genetic similarity of the IuMien with other East Asian populations is admixture as indicated by mixed languages between the IuMien and Sinitic languages [64]. 

### 4.2. The Genetic Structure of the Lahu and Their Linguistic Relatives, Lisu and Akha

There are two main ST subfamilies: Chinese and Tibeto-Burman, which have been separated around 6 thousand years ago (kya) based on lexical data [65]. The putative ancestors of the modern ST populations are either the Neolithic people living at least 6 kya in northwestern China [66] or the millet farmers, located in North China, around 7.2 kya [67] or 5.9 kya [68]. Within the Tibeto-Burman language, both linguistic and genetic studies indicate differences between the Tibetan and Lolo-Burmese (or Ngwi-Burmese) languages [67,68,69,70]. There are four ST groups in this study: Akha, Lisu, Lahu and Karen. The languages of Lahu Akha and Lisu belong to the Lolo-Burmese, while the Karen speak the Karenic branch [3]. Here, we focus to discuss the Lahu, Akha and Lisu who have been less studied than the Karen.

Based on the historical evidence, the Akha Lisu and Lahu migrated from southern China through Myanmar to northern Thailand about 100–200 years ago [1]. Although bearing less distinction from the other groups than the Hmong, the Lahu, based on our results, exhibit genetic difference from other Thai populations (Figure 3). In agreement with the previous mtDNA and Y chromosomal study [12], as a result of isolation by genetic drift that promoted their differentiation, both populations of the Lahu have prevalent haplogroups: haplogroup F for Y chromosome and haplogroup D4j1a1 and G1c for mtDNA. Previous studies also revealed that the Thai and Vietnamese Lahu show relatedness in the paternal side but not in the maternal side [12] and the Thai and Chinese Lahu are genetically similar [18]. Although the Akha and Lisu do differ significantly in terms of genetic distance (Figure 2) from other populations, the MDS and STRUCTURE results show similarity with the other populations (Figure 3 and Figure 4), suggesting interactions between the Lisu and Akha and other populations. The previous results also supported interaction between the Lisu and other populations [12,18] indicative of mixed ancestries of the Lisu and Akha probably due to Sinicization in southern China before movement to Thailand [1].

### 4.3. The Genetic Structure of the Htin and Their Linguistic Relatives, Khmu and Mlabri

The languages of the Htin, Khmu and Mlabri belong to the Khmuic branch of the AA family. Composed of two subgroups of Mal and Pray, the Htin migrated from their homeland in Laos to northern Thailand at the turn of the 20th century A.D. [1,3]. With a nomadic traditional lifestyle and census size ~400 individuals [1,3], the Mlabri migrated from Laos to northern Thailand during the 19th century A.D. Our result indicated an extremely reduced genetic diversity, i.e., no variation at *TPOX* (*H_E_* = 0) and only 51 alleles in total (Table 1), possibly driven by genetic drift associated with isolation and very small population sizes. Previous genetic studies based on mtDNA, Y chromosome, and autosomal diversity supported strong genetic drift of the Mlabri [17,20,71] and genetic relatedness between the Htin and Mlabri. More specifically, both the Htin Mal and Pray have genetic clustering with the Mlabri in the paternal but not in the maternal side, indicating contrasting male and female genetic variations [20]. The present result indicates that the Htin and Mlabri are standouts in their genetic structures (Figure 4), while unexpectedly the Khmu have close relatedness to many TK and ST populations (Figure 3) and share ancestry with them (Figure 4). Although the Khmu are also one of the oldest inhabitants in northern Laos and northern Thailand, the main homeland of the Khmu sampled for this study is in Northern Laos, from where the Htin Mal and Htin Pray and Mlabri also originated. The Khmu have migrated back and forth across the border of Thailand and Laos until present day. Because Khmu villages are located in lower elevation of the hills of northern Thailand along the Laotian border and they conduct business with or through the Thais, extensive contacts between the Thai Khmu and Thais and intermarriage with the Laotian have been recorded [72]. Previous genome-wide data supported sharing between the Khmu and Laotian [18], in line with the mtDNA shared haplotypes among both groups but in contrast with the high divergence of the Khmu from their Y chromosomal profiles [11].

### 4.4. The Genetic Structure of the Palaung and Their Linguistic Relatives, Blang and Lawa 

Unlike the hill tribe Lawa who are regarded as native to northern Thailand, the migrant Palaung and Blang are highlanders and minority groups in northern Thailand who are not officially recognized as hill tribes. However, despite the difference in official recognition, all of them speak the same language branch of Palaungic, which is the most northerly spoken AA language, spanning northern Thailand, northern Laos, northern Myanmar and southern China. The first group of the Palaung recently moved from the Shan State of Myanmar to northern Thailand about 40 years ago, while the Blang migrated from southern China through Myanmar to Thailand about 60 years ago [1]. Genetic difference of the Palaung from the other AA populations observed in this study (Figure 3) is consistent with the previous Y chromosomal result [11] in which the Palaung exhibited genetic relatedness to the ST-speaking populations. Because the ancestor of the Palaung settled in the upper part of the Salween River long before the arrival of most other ethnic groups in Myanmar [1], genetic admixture with several ST-speaking groups, e.g., Burmese, could promote their affinity. In contrast, both Blang populations show genetic similarity to the TK and ST populations (except Lahu) (Figure 3), although genetic heterogeneity within the Blang 2 was observed (Figure 4). In general, the three Lawa populations are clustered with the TK and most ST populations (Figure 3), in agreement with previous studies showing the interaction between Lawa and ST-speaking Karen, e.g., sharing Y chromosomal haplotypes [12] and ancestry [18]. However, in the STRUCTURE result at *K* = 10, the Lawa Western stand out from the Lawa Eastern who still share a component with the TK groups, supported by historical record that indicated contact of the Lawa Eastern with the northern Thai TK group [73].

## 5. Conclusions

Previous investigations of forensic STRs in Thailand were conducted on major lowland groups from all regions but complete forensic database of the hill tribes were lacking. In this work, we generated autosomal forensic STRs data of all hill tribes and some non-hill tribe groups from several villages in northern Thailand, constructed an allelic frequency table and explored genetic relationship among them. In general, we found genetic divergence of the Hmong from other populations, genetic difference between the Hmong and IuMien, and genetic heterogeneity of the AA and ST groups mirroring various population interactions that were possibly driven by geographic proximity and previous admixture events. The complete forensic STRs data for the hill tribes here provide advantages for further forensic investigation in Thailand and would also benefit Laos and Myanmar wherein large populations of the many ethnicities investigated in this study still reside. In addition, we also explored the genetic diversity, migration and demographic history of the northern Thai highlanders.

## Figures and Tables

**Table 1:** General information of studied populations, genetic diversity indices and forensic parameters.

Ethnicity	Populations	Sample Size	Language	References	Gene Diversity (SD)	Average *H_E_*	Total Allele	CMP	CPD	CPE	Loci Departed from HWE
Akha	Akha	38	Sino-Tibetan	Present study	0.766 (0.390)	0.773	110	1.28 × 10^−15^	0.9999888	0.999999999999999	
Lahu	Lahu Black	25	Sino-Tibetan	Present study	0.729 (0.373)	0.735	94	1.02 × 10^−13^	0.9999955	0.999999999999898	
	Lahu Red	24	Sino-Tibetan	Present study	0.707 (0.363)	0.715	91	5.88 × 10^−13^	0.9999930	0.999999999999412	
Karen	Karen Skaw	23	Sino-Tibetan	Present study	0.762 (0.390)	0.767	97	5.67 × 10^−14^	0.9999942	0.999999999999943	
	Keren Kayah	46	Sino-Tibetan	Kutanan et al. (2015)	0.752 (0.381046)	0.752	103	4.58 × 10^−15^	0.9999984	0.999999999999995	
Lisu	Lisu	26	Sino-Tibetan	Present study	0.759 (0.388)	0.761	100	1.58 × 10^−14^	0.9999884	0.999999999999984	
Hmong	Hmong Njua 1	58	Hmong–Mien	Present study	0.719 (0.365)	0.721	111	1.76 × 10^−14^	0.9999462	0.999999999999982	
	Hmong Daw 1	21	Hmong–Mien	Present study	0.717 (0.371)	0.726	91	3.29 × 10^−13^	0.9999813	0.999999999999671	
	Hmong Njua 2	29	Hmong–Mien	Present study	0.736 (0.376)	0.740	102	5.39 × 10^−14^	0.9999968	0.999999999999946	
	Hmong Daw 2	32	Hmong–Mien	Present study	0.746 (0.388)	0.764	117	4.83 × 10^−15^	0.9999886	0.999999999999995	*vWA*
	Hmong Njua 3	17	Hmong–Mien	Present study	0.720 (0.372)	0.720	81	7.16 × 10^−12^	0.9999871	0.999999999992835	*FGA*
IuMien	IuMien	35	Hmong–Mien	Present study	0.761 (0.392)	0.767	111	5.42 × 10^−15^	0.9999113	0.999999999999995	*D18S51*
Mlabri	Mlabri	19	Austro-Asiatic	Present study	0.547 (0.288)	0.5470	51	8.83 × 10^−9^	0.9986837	0.999999991173593	
Htin	Htin Mal	37	Austro-Asiatic	Present study	0.719 (0.366)	0.733	108	6.47 × 10^−14^	0.9999270	0.999999999999935	*D19S433*
	Htin Pray 1	26	Austro-Asiatic	Present study	0.723 (0.370)	0.739	92	1.52 × 10^−13^	0.9999562	0.999999999999848	*TH01*, *D18S51*
	Htin Pray 2	41	Austro-Asiatic	Present study	0.765 (0.388)	0.765	106	1.8 × 10^−15^	0.9999912	0.999999999999998	
Khmu	Khmu	26	Austro-Asiatic	Present study	0.737 (0.379)	0.749	95	3.94 × 10^−14^	0.9999682	0.999999999999961	*FGA*
Lawa	Lawa Western 1	39	Austro-Asiatic	Present study	0.752 (0.385)	0.768	108	5.31 × 10^−15^	0.9999816	0.999999999999995	
	Lawa Western 2	47	Austro-Asiatic	Kutanan et al. 2011)	0.751 (0.381)	0.753	103	3.82 × 10^−15^	0.9999987	0.999999999999996	
	Lawa Eastern	50	Austro-Asiatic	Kutanan et al. (2011)	0.767(0.388)	0.767	114	8.99 × 10^−16^	0.9999996	0.999999999999999	
Palaung	Palaung	54	Austro-Asiatic	Present study	0.747 (0.378)	0.754	119	2.71 × 10^−15^	0.9999782	0.999999999999997	
Blang	Blang 1	35	Austro-Asiatic	Present study	0.788 (0.400)	0.797	125	5.72 × 10^−16^	0.9999717	0.999999999999999	
	Blang 2	27	Austro-Asiatic	Present study	0.774 (0.395)	0.776	118	2.00 × 10^−15^	0.9999930	0.999999999999998	
Shan	Shan 1	44	Tai-Kadai	Kutanan et al. (2011)	0.783 (0.396)	0.783	117	5.83 × 10^−16^	0.9999939	0.999999999999999	
	Shan 2	22	Tai-Kadai	Present study	0.762 (0.390)	0.768	110	2.68 × 10^−14^	0.9999996	0.999999999999973	
Yuan	Yuan	87	Tai-Kadai	Kutanan et al. (2011)	0.781 (0.393)	0.781	126	7.16 × 10^−17^	0.9999973	0.999999999999999	
Yong	Yong	55	Tai-Kadai	Kutanan et al. (2011)	0.776 (0.392)	0.734	125	5.23 × 10^−16^	0.9999974	0.999999999999999	

* indicate statistical significance at *p* < 0.05.

**Table 2:** Analysis of molecular variance (AMOVA) result.

	No. of Groups	No. of Populations	% of Variance		
			Within Populations	Among Populations within Groups	Among Groups
All studied sample	1	27	96.09	3.91 *	
Sino-Tibetan (ST)	1	6	96.69	3.31 *	
Hmong–Mien (HM)	1	6	98.61	1.39 *	
Austroasiatic (AA)	1	11	95.07	4.93 *	
Austroasiatic (excluding the Mlabri)	1	10	96.27	3.73 *	
Tai-Kadai (TK)	1	4	99.46	0.54 *	
ST/HM/AA/TK	4	27	95.83	3.11 *	1.06 *
AA vs. ST	2	17	94.95	4.46 *	0.59 *
AA vs. TK	2	12	96.35	3.79	−0.14
AA vs. HM	2	14	93.67	3.87	2.45 *
HM vs. TK	2	10	97.28	0.83 *	1.90 *
HM vs. ST	2	12	95.20	2.37 *	2.42 *
ST vs. TK	2	10	98.02	1.95 *	0.03

* indicate statistical significance at *p* < 0.05.

## Data Availability

Raw genotyping data of 654 individuals are provided in Table S1.

## Supplementary Materials

The following are available online at https://www.mdpi.com/2073-4425/12/3/383/s1, Figure S1: Number of populations with the highest posterior probability expressed as the Delta K; Table S1: Newly generated raw genotyping data in allele; Table S2: Allelic frequency and forensic parameters of all hill tribes (Akha, Lahu, Karen, Lisu, Hmong, IuMien, Htin, Khmu and Lawa) (*n* = 640); Table S3: Allelic frequency and forensic parameters of highlanders including hill tribes and non hill tribes (Akha, Lahu, Karen, Lisu, Hmong, IuMien, Htin, Khmu, Lawa, Mlabri, Paluang, Shan and Blang) (*n* = 841); Table S4: Allelic frequency and forensic parameters of the Akha (*n* = 38); Table S5: Allelic frequency and forensic parameters of the Lahu (*n* = 49); Table S6: Allelic frequency and forensic parameters of the Karen (*n* = 69); Table S7: Allelic frequency and forensic parameters of the Lisu (*n* = 26); Table S8: Allelic frequency and forensic parameters of the Hmong (*n* = 157); Table S9: Allelic frequency and forensic parameters of the IuMien (*n* = 35); Table S10: Allelic frequency and forensic parameters of the Mlabri (*n* = 19); Table S11: Allelic frequency and forensic parameters of the Htin (*n* = 123); Table S12: Allelic frequency and forensic parameters of the Khmu (*n* = 26); Table S13: Allelic frequency and forensic parameters of the Lawa (*n* = 136); Table S14: Allelic frequency and forensic parameters of the Paluang (*n* = 54); Table S15: Allelic frequency and forensic parameters of the Blang (*n* = 62); Table S16: Allelic frequency and forensic parameters of the Shan (*n* = 66).
